# Utilizing CT imaging for evaluating late gastrointestinal tract side effects of radiotherapy in uterine cervical cancer: a risk regression analysis

**DOI:** 10.1186/s12880-024-01420-3

**Published:** 2024-09-09

**Authors:** Pooriwat Muangwong, Nutthita Prukvaraporn, Kittikun Kittidachanan, Nattharika Watthanayuenyong, Imjai Chitapanarux, Wittanee Na Chiangmai

**Affiliations:** 1 Division of Radiation Oncology, Department of Radiology, Faculty of Medicine, Chiang Mai University, Chiang Mai, Thailand; 2 Division of Diagnostic Radiology, Department of Radiology, Faculty of Medicine, Chiang Mai University, Chiang Mai, Thailand

**Keywords:** Cervical cancer, Radiotherapy, Toxicity, CT, Gastrointestinal

## Abstract

**Background:**

Radiotherapy (RT) is effective for cervical cancer but causes late side effects (SE) to nearby organs. These late SE occur more than 3 months after RT and are rated by clinical findings to determine their severity. While imaging studies describe late gastrointestinal (GI) SE, none demonstrate the correlation between the findings and the toxicity grading. In this study, we demonstrated the late GI toxicity prevalence, CT findings, and their correlation.

**Methods:**

We retrospectively studied uterine cervical cancer patients treated with RT between 2015 and 2018. Patient characteristics and treatment(s) were obtained from the hospital’s databases. Late RTOG/EORTC GI SE and CT images were obtained during the follow-up. Post-RT GI changes were reviewed from CT images using pre-defined criteria. Risk ratios (RR) were calculated for CT findings, and multivariable log binomial regression determined adjusted RRs.

**Results:**

This study included 153 patients, with a median age of 57 years (IQR 49–65). The prevalence of ≥ grade 2 RTOG/EORTC late GI SE was 33 (27.5%). CT findings showed 91 patients (59.48%) with enhanced bowel wall (BW) thickening, 3 (1.96%) with bowel obstruction, 7 (4.58%) with bowel perforation, 6 (3.92%) with fistula, 0 (0%) with bowel ischemia, and 0 (0%) with GI bleeding. Adjusted RRs showed that enhanced BW thickening (RR 9.77, 95% CI 2.64–36.07, *p* = 0.001), bowel obstruction (RR 5.05, 95% CI 2.30–11.09, *p* < 0.001), and bowel perforation (RR 3.82, 95% CI 1.96–7.44, *p* < 0.001) associated with higher late GI toxicity grades.

**Conclusions:**

Our study shows CT findings correlate with grade 2–4 late GI toxicity. Future research should validate and refine these findings with different imaging and toxicity grading systems to assess their potential predictive value.

## Introduction

Radiotherapy (RT) stands as a common and effective approach for treating uterine cervical cancer. It serves as both a post-surgery option for patients with unfavorable pathological characteristics and as a primary treatment [1–3]. Despite advancements in radiotherapy that enable precise targeting of radiation to specific areas, nearby healthy organs inevitably receive some portion of the radiation dose, leading to side effects that affect these neighboring organs [4–6].

Late side effects of RT refer to the consequences as a result of radiation therapy that occur more than three months after irradiation [7]. These consequences are primarily attributed to ischemia and fibrotic alterations of normal organs [8]. In the gastrointestinal (GI) system, a spectrum of toxicities arises, spanning from mild forms like enteritis, intestinal wall fibrosis, and telangiectasia to severe manifestations including ulcers, hemorrhages, strictures, fistulas, and perforations. Clinical manifestations can vary and encompass symptoms such as abdominal pain, diarrhea, nausea, vomiting, flatulence, weight loss, and bowel obstruction [4–6, 9–11]. The assessment of organ toxicity severity typically relies on the evaluation of patient symptoms, clinical measurements, and therapy interventions [12–14].

Imaging is important for evaluating late GI toxicity [6, 15]. Several studies have demonstrated image-related alterations in GI organs receiving radiotherapy. These image findings include bowel wall thickening, strictures, tethering, small bowel obstruction, perforation, and fistula formation, all of which can be identified in patients following radiotherapy [16–20].

In this context, our study explores the potential utility of CT findings as indicators for predicting late grade 2–4 GI toxicity in patients with cervical cancer treated with RT. By examining the prevalence of late GI side effects, the occurrence of CT findings associated with GI toxicities, and the correlation between these findings and late GI side effects, we aim to offer an understanding of the role of imaging in assessing the late GI side effect of radiotherapy. Through this investigation, we aim to contribute insights into the potential integration of CT findings as a supplement to conventional clinical evaluations in determining treatment-related toxicities.

## Materials and methods

A retrospective observational cohort study was undertaken to examine the correlation between CT findings and GI late adverse effects in patients with uterine cervical cancer who underwent radiotherapy at Maharaj Nakorn Chiang Mai Hospital in Thailand from January 2015 to December 2018. The inclusion criteria were: (1) a confirmed histological diagnosis of uterine cervical cancer at FIGO 2018 stages IA1-IVA, excluding small cell carcinoma, malignant melanoma, and cervical sarcoma; (2) treatment with radiotherapy (RT) using conventional doses per fraction of external beam RT, with or without brachytherapy, following surgery or as definitive curative treatment; (3) a minimum follow-up period of three months post-RT; and (4) availability of at least one CT image captured no less than three months after RT.

Baseline patient characteristics, treatment details, and grading of late GI tract toxicity were obtained from the radiation oncology database and hospital medical records, using the RTOG/EORTC late toxicity criteria. CT images were retrieved from the hospital’s Picture Archiving and Communication System (PACS). The FIGO staging was updated to reflect the 2018 FIGO staging classification.

This study adhered to the principles of the Helsinki Declaration and was granted approval by our institute’s Ethical Committee under number 499/2021.

### RT, chemotherapy, and follow-up

For definitive RT, 50 Gy (Gy) of whole pelvic RT (WPRT) was prescribed. In the cases of paraaortic lymph node or tumor involvement of the lower one-third of the vagina, RT fields were extended to include the paraaortic lymph node (PAN) area or bilateral inguinal lymph node area, respectively. In the final week of external-beam RT, a four-session brachytherapy boost of 7 Gy per session was initiated.

In the postoperative setting, 50 Gy of WPRT was prescribed. Brachytherapy was administered to patients with a positive vaginal margin.

Either weekly cisplatin 40 mg/m^2^ or weekly carboplatin AUC2 was administered concurrently with RT in patients with FIGO stages IB3, IIA2, IIB, IIIC1, and IIIC2 receiving definitive RT, as well as those who had undergone surgery and had positive surgical margins, lymph node metastases, or parametrial invasion.

Following the completion the treatment, patients were evaluated for clinical response though per vaginal examination and treatment toxicities were assessed according to RTOG/EORTC late toxicity criteria [13]. Evaluations were conducted every 3 months for the first year, every 4 months for the second year, every 6 months for the next 2 years, and then annually. The following criteria were used to evaluate late GI toxicity during the follow-up: grade 0 – none; grade 1 – mild diarrhea, mild cramping, bowel movement 5 times daily, slight rectal discharge or bleeding; grade2 – moderate diarrhea and colic, bowel movement > 5 times daily, excessive rectal mucus or intermittent bleeding; grade 3 – obstruction or bleeding, requiring surgery; grade 4 – necrosis / perforation fistula; and grade 5 – death related to radiation late effects.

Within the framework of this study, late GI toxicity was categorized into two groups for analysis: grade 0–1 group and grade 2–5 group.

### CT image assessment

CT images of the pelvis or the whole abdomen were used to assess tumor response in patients with initial pelvic or paraaortic nodal metastasis, as well as to evaluate those suspected of having recurrent or persistent disease. Additionally, it is employed to assess the toxicity of radiotherapy in individuals showing symptoms.

All CT scans were carried out with multidetector CT scanners and intravenous contrast media. The axial images of abdomen and pelvic cavity in the portal venous phase were performed after injection of 100–150 ml of iodinated contrast media (320–350 mg of iodine per milliliter) with flow rate of 3–5 ml/sec. Axial images were reconstructed at 2-mm and 5-mm thickness. Multiplanar reconstruction comprising coronal and sagittal images were created at a 3-mm thickness.

For this study, CT image acquisition within one month of the clinical evaluation of late toxicities in follow-up assessments was selected. When multiple CT images were available, we chose to evaluate the most recent scan that showed the highest grade of late GI toxicity.

**Fig. 1:**
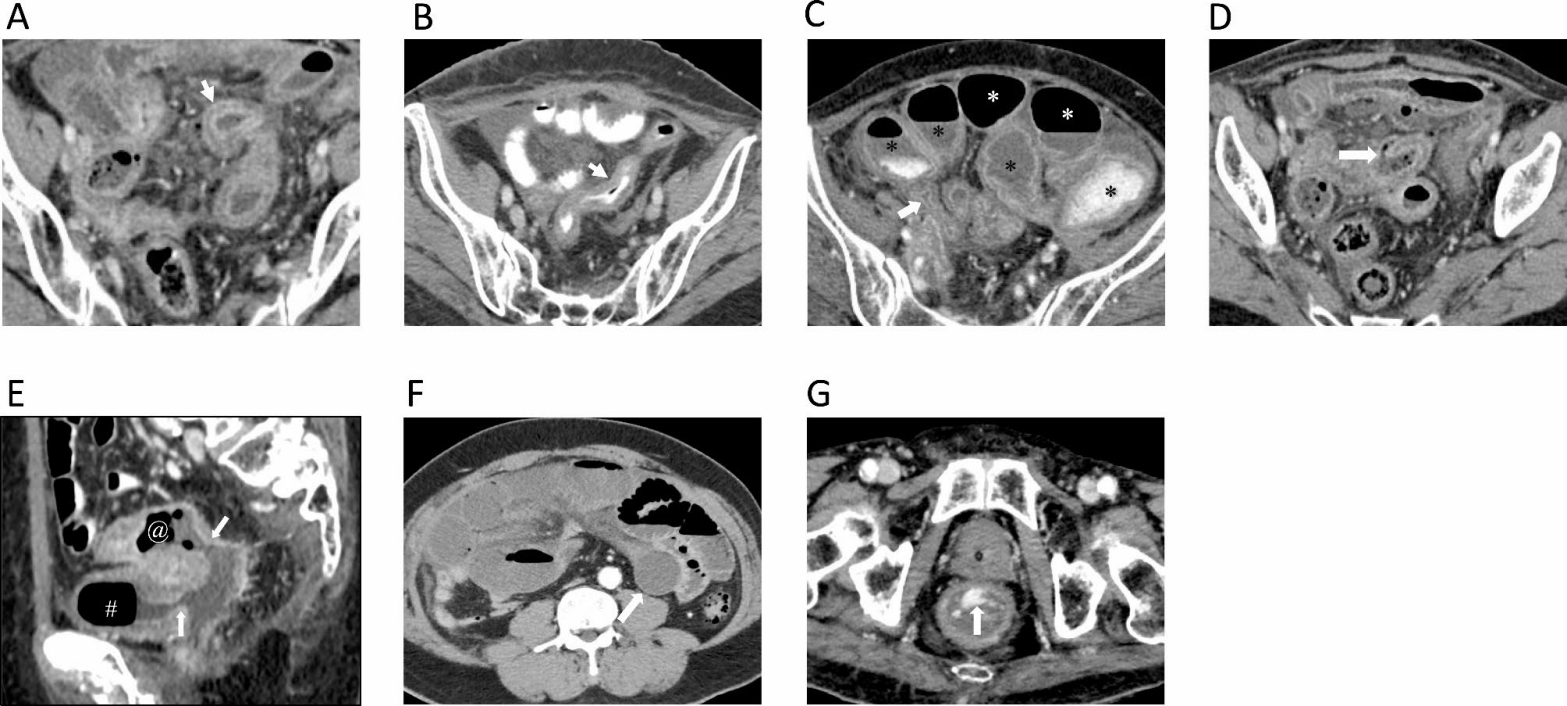
CT findings of radiation-induced late gastrointestinal toxicity. (**A**) Bowel wall thickening with target water bowel wall enhancement in distended bowel loop (arrow); (**B**) Bowel wall thickening with isoattenuation bowel wall enhancement in collapsed bowel loop (arrow); (**C**) Bowel obstruction; dilatation of the bowel loops [*] with transitional point (arrow); (**D**) Bowel wall disruption (arrow) in bowel perforation; (**E**) Sagittal CT shows fistula formation (arrow), connection between small bowel [@] and urinary bladder [#]; (**F**) Axial CT shows non-enhancing bowel wall (arrow) suggestive of intermediate to late-stage bowel ischemia. (**G**) Axial CT shows contrast extravasation into the rectal lumen (arrow)

An experienced radiologist with board certification and a trainee in their third year of a diagnostic radiology residency program jointly reviewed the axial CT images from the portovenous phase. They conducted the review in consensus and without access to clinical data, focusing on the CT findings that followed:

- *Enhanced bowel-wall thickening*, defined as single wall thickness exceeding 3 mm in distended loops and exceeding 5 mm in collapsed loops [20] (Fig. 1A and B).
- *Bowel obstruction* was defined as upstream dilated bowel loops (greater than 2.5 cm in small bowel and greater than 6 cm in large bowel) with transitional point [20] (Fig. 1C).
- *Bowel perforation* was defined as bowel wall disruption along the mucosa to serosa or presence of pneumoperitoneum [18] (Fig. 1D).
- *Fistula formation* was defined as presence of connection between lumen of the bowel loops to the lumen of the adjacent organs such as another bowel loop, bladder, uterus, vaginal or skin [17] (Fig. 1E).
- *Bowel ischemia* was defined as transmural hyper-enhancement suggestive of early ischemia and hypo-enhancing or non-enhancing bowel wall suggestive of intermediate to late-stage bowel ischemia (Fig. 1F).
- (f) *GI bleeding* was defined as contrast extravasation into the intestinal lumen (Fig. 1G).

### Statistical analysis

Based our pivot data, we determined that the highest number of samples originated from cases of fistula formation in late GI toxicity in CT findings graded as 0–1 and 2–4, with prevalence of 2% and 10%, respectively. With a power of 0.8 and a significance level of 0.05, our study required a sample size of 138.

Patient characteristics, treatments, late GI toxicity, and CT findings were summarized using descriptive statistics. Quantitative data were presented as medians with interquartile ranges (IQR), while categorical data were expressed as numbers with corresponding percentages. To assess group differences, the Wilcoxon rank-sum test was employed for quantitative variables, while Fisher’s exact test was used for categorical variables. Risk ratios were computed for CT findings, and further risk ratios, adjusted for patient age, chemotherapy regimen, radiotherapy technique, treatment fields, brachytherapy, histology, and treatment objective, were determined using a multivariable log binomial regression with a Poisson working model. Statistical significance was set at *p* < 0.05. All analyses were conducted using STATA software version 16 (Stata Corp LLC, Texas, USA).

## Results

This study included 153 eligible patients with a median age of 57 years (IQR 49–65). The most prevalent tumor stages were IIB (51 patients, 33.33%), IIIB (45 patients, 29.41%), and IIIC2 (19 patients, 11.11%). Radiation techniques consisted of 84 cases of conventional (54.90%), 39 cases of three-dimensional conformal RT (3D-CRT) (25.49%), and 30 cases of intensity modulated radiation therapy (IMRT) (19.61%). The radiation fields encompassed WPRT alone in 124 cases (81.05%), WPRT with PAN in 16 cases (10.46%), WPRT with inguinal area in 10 cases (6.54%), and WPRT with both PAN and inguinal area in 3 cases (1.96%). Brachytherapy was administered to 136 patients (88.89%). Chemotherapy was administered to 127 patients (82.81%), consisting of cisplatin in 121 patients and carboplatin in 5 patients. The treatment setting was definitive for 140 (91.50%) patients and post-operative for 13 (8.50%) patients. Except for brachytherapy, patient characteristics and treatments were comparable between the RTOG/EORTC late GI toxicity grade 0–1 group and the grade 2–4 group. (Table 1)

**Table 1:** Patient characteristics

Parameter	Grade 0–1 (*n* = 120)	Grade 2–4 (*n* = 33)	*P*-value
**Median Age**, **years (IQR)**	56.5 (49–64)	60 (50–67)	0.649
**Median F/U**, **years (IQR)**	3.01 (1.56–4.21)	2.27 (1.37–3.85)	0.1630
**FIGO staging (%)**			
IB1 IB2 IB3 IIA2 IIB IIIA IIIB IIIC1 IIIC2 IVA	2 (1.67) 2 (1.67) 1 (0.83) 5 (4.17) 40 (33.33) 3 (2.50) 36 (30.00) 13 (10.83) 14 (11.67) 4 (3.33)	0 (0.00) 0 (0.00) 1 (3.03) 0 (0.00) 11 (33.33) 0 (0.00) 9 (27.27) 1 (3.03) 5 (15.15) 6 (18.18)	0.159
**Pathology (%)**			
SCCA Adenocarcinoma Adenosquamous	103 (85.83) 14 (11.67) 3 (2.50)	31(93.94) 1 (3.03) 1 (3.03)	0.312
**Treatment aim (%)**			
Definitive Post-operative	108 (90.00) 12 (10.00)	32 (96.97) 1 (3.03)	0.301
**Chemotherapy (%)**			
Cisplatin Carboplatin None	97 (80.83) 4 (3.44) 19 (15.83)	24 (72.73) 1 (3.03) 8 (24.24)	0.458
**Radiotherapy technique (%)**			
Conventional 3D-CRT IMRT	67 (55.83) 28 (23.33) 25 (29.83)	17 (51.52) 11 (33.33) 5 (15.15)	0.484
**Field (%)**			
WPRT WPRT + PAN WPRT + Ing WPRT + PAN + Ing	100 (83.33) 13 (10.83) 6 (5.00) 1 (0.83)	24 (72.73) 3 (9.09) 4 (12.12) 2 (6.06)	0.101
**Brachytherapy (%)**	103 (85.83)	33 (100.00)	0.024

F/U: follow-up; CT: computed tomography; SCCA: squamous cell carcinoma; 3D-CRT: three-dimensional conformal radiotherapy; IMRT: intensity modulated radiation therapy; WPRT: whole-pelvic radiotherapy; PAN: paraaortic nodal radiotherapy; Ing: inguinal radiotherapy

The incidence of RTOG/EORTC late GI toxicity grade 0 was observed in 110 patients (71.90%), while grades 1, 2, 3, and 4 toxicities were reported in 10 (6.54%), 13 (8.50%), 14 (9.15%), and 6 (3.92%) patients, respectively. No grade 5 toxicities were recorded.

CT findings revealed that out of the total number of 153 patients, 91 patients (59.48%) had enhanced thickened bowel walls, 3 (1.96%) had bowel obstruction, 7 (4.58%) had bowel perforation, 6 (3.92%) had fistula, 0 (0%) had bowel ischemia, and 0 (0%) had GI bleeding. A comparison of positive CT findings between the grade 0–1 and grade 2–4 toxicity groups is presented in Table 2. The outcomes demonstrated significant differences between the two groups for enhanced bowel wall thickening, bowel obstruction, and bowel perforation, but not for fistula formation.

**Table 2:** Comparison between CT findings and late GI toxicity grading

CT Imaging finding	Positive finding, *N* (%)		*P*-value
	Grade 0–1 (*n* = 120)	Grade 2–4 (*n* = 33)	
Enhanced bowel wall thickening	60 (50)	31 (93.94)	< 0.001
Bowel obstruction	0 (0)	3 (9.09)	0.009
Bowel perforation	1 (0.83)	6 (18.18)	< 0.001
Fistula formation	3 (2.50)	3 (9.09)	0.115
Bowel ischemia	0 (0)	0 (0)	N/A
GI bleeding	0 (0)	0 (0)	N/A

Table 3 shows the risk ratios of CT findings, excluding bowel ischemia and GI bleeding, as these did not yield any positive findings in this study. Risk ratios of all CT findings, except for fistula formation, were significant. These outcomes suggest a higher likelihoods of higher grade 2–4 late GI toxicity in cases with positive CT findings compared those with negative findings. After multivariable analysis, adjusting for variables including age, chemotherapy regimen, radiotherapy technique, treatment fields, brachytherapy, histology, and treatment objective, the results consistently indicated an elevated risk of grade 2–4 late GI toxicity in patients with positive CT findings across all categories, except for fistula formation.

**Table 3:** Risk ratio

CT findings	Univariate		Multivariate*	
	Risk ratio (95% CI)	*P*-value	Risk ratio (95% CI)	*P*-value
Enhanced bowel wall thickening	10.56 (2.62–42.52)	0.001	9.77 (2.64–36.07)	0.001
Bowel obstruction	5.00 (3.63–6.89)	< 0.001	5.05 (2.30-11.09)	< 0.001
Bowel perforation	4.63 (2.94–7.31)	< 0.001	3.82 (1.96–7.44)	< 0.001
Fistula formation	2.45(1.04–5.80)	0.084	0.71 (0.24–2.13)	0.540

* Adjusted for age, chemotherapy regimen, radiotherapy technique, treatment fields, brachytherapy, histology, and treatment aim

## Discussion

While conventional grading systems primarily rely on patients’ reported symptoms and the treatments they receive to assess the severity of toxicities [12–14], our study revealed CT findings can also serve as an additional determinant for grade 2–4 toxicity. Specifically, our research highlighted that CT findings, namely enhanced thickened bowel walls, bowel obstruction, and bowel perforation were linked to more severe late GI toxicity. These CT findings help in determining severity of the GI toxicity.

In our study, we found that enhanced bowel wall thickening, bowel obstruction, and bowel perforation were the three CT findings significantly associated with a higher grade of late GI toxicity. Among these three findings, enhanced bowel wall thickening exhibited the most substantial impact in predicting grade 2–4 toxicity compared to those who had negative findings, demonstrating a relative risk (RR) of 10.56. This was followed by bowel obstruction, with an RR of 5.0, and bowel perforation, with an RR of 4.63. These outcomes remained consistent even after multivariate analysis, which adjusted for patient characteristics and treatment factors, yielding respective RRs of 9.77, 5.05, and 3.82.

Our study also unveiled that enhanced bowel wall thickening was the most prevalent finding, observed in over half of the patients, comprised of 50% in late GI toxicity grade 0–1 group and 93.94% in grade 2–4 group. This can be attributed to the pathophysiological alterations in the irradiated bowel wall, leading to increased collagen deposition and subsequent thickening and immobilization of the bowel loop [6]. However, even with lower prevalent findings of bowel obstruction and bowel perforation, these findings are more likely to prompt management suggesting clinically significant of these findings.

Our findings indicated that using fistula formation as an indicator for evaluating grade 2–4 toxicity yielded negative results. Additionally, we identified one patient with bowel obstruction who was classified in the toxicity grade 0–1 group. These results highlight the limitations of relying solely on clinical assessment for evaluating late GI toxicity. If these patients undergone both CT imaging and clinical evaluation, it becomes clear that their treatment related to CT findings might have resulted in a shift in their toxicity grading, potentially raising them to grade 3 or 4. These results emphasize the advantages of incorporating CT imaging into the follow-up process, rather than relying solely on clinical evaluation. This approach is in line with current guidelines that advocate for the inclusion of imaging during follow-up [2, 3].

Despite the highest RR of 9.77 observed in cases of enhanced thickened bowel wall, which implies that patients with positive CT findings in this category are nearly ten times more likely to experience grade 2–4 late GI toxicity than those with negative findings, our findings revealed that half of the patients categorized under grade 0–1 toxicity exhibited positive findings. Given that prior research has highlighted the tendency for physician-reported toxicities to underestimate the true impact when compared to patient-reported outcomes [21–24], it becomes essential to place special emphasis on individuals presenting with an enhanced thickened bowel wall. Ensuring that these patients do not experience GI symptoms is of paramount importance, as any indication of symptoms should trigger prompt treatment [4].

To the best of our knowledge, our study is the first to demonstrate the correlation of CT findings and late grade 2–4 GI toxicity in cervical cancer. We used basic CT scan results and highlighted how each result can predict the later GI toxicity. This approach could become a regular component of patient care.

There were limitations in our study. Firstly, the retrospective nature of our study introduces potential biases and confounding. Secondly, our study exclusively utilized CT images and assessed late GI toxicity based solely on RTOG/EORTC late toxicity criteria, focusing only on cervical cancer. These factors may limit generalizability of our results to other imaging modalities, alternative grading systems, or other malignancies requiring pelvic irradiation, such as endometrial cancer, where treatment protocols differ and vary based on surgical pathology and molecular classification [25, 26]. Thirdly, our study relied solely on binary outcomes derived from CT findings, potentially overlooking specific details within the findings.

Our study demonstrated the potential for incorporating CT findings into the late GI toxicity assessment for refining severity categorization beyond conventional grading systems. Integrating CT imaging into follow-up protocols could enhance the accuracy of late GI toxicity evaluation. Further investigations should explore alternative imaging modalities, such as CT enterography or MR enterography, and consider using alternative toxicity grading systems like CTCAE to validate our findings. Additionally, the study of radiomic features in conjunction with other malignancies requiring pelvic irradiation may provide advantages in finely assessing treatment toxicity. Prospective studies are essential to validate and enhance the robustness of our current findings.

## Conclusion

Our study indicates that CT findings, particularly enhanced thickened bowel wall, bowel obstruction, and bowel perforation, are correlated with grade 2–4 late GI toxicity. While acknowledging the retrospective design and inherent limitations, this approach could enhance the assessment of treatment-related side effects. Further research incorporating different imaging modalities and toxicity grading systems is warranted to validate our findings and to assess their potential predictive capability.

## Data Availability

The data that support the findings of this study are available from the corresponding author upon reasonable request. The data are not publicly available due to containing information that could compromise research participant privacy.
